# The mitochondrial alternative oxidase Aox1 is needed to cope with respiratory stress but dispensable for pathogenic development in *Ustilago maydis*

**DOI:** 10.1371/journal.pone.0173389

**Published:** 2017-03-08

**Authors:** Christian A. Cárdenas-Monroy, Thomas Pohlmann, Gabriela Piñón-Zárate, Genaro Matus-Ortega, Guadalupe Guerra, Michael Feldbrügge, Juan Pablo Pardo

**Affiliations:** 1 Departamento de Bioquímica, Facultad de Medicina, UNAM. Ciudad de México, México; 2 Institute for Microbiology, Cluster of Excellence on Plant Sciences, Department of Biology, Heinrich-Heine University Düsseldorf, Düsseldorf, Germany; 3 Departamento de Biología Celular y Tisular, Facultad de Medicina, UNAM. Ciudad de México, México; 4 Departamento de Microbiología, Escuela Nacional de Ciencias Biológicas, Instituto Politécnico Nacional. Ciudad de México, México; Universidade Federal de Vicosa, BRAZIL

## Abstract

The mitochondrial alternative oxidase is an important enzyme that allows respiratory activity and the functioning of the Krebs cycle upon disturbance of the respiration chain. It works as a security valve in transferring excessive electrons to oxygen, thereby preventing potential damage by the generation of harmful radicals. A clear biological function, besides the stress response, has so far convincingly only been shown for plants that use the alternative oxidase to generate heat to distribute volatiles. In fungi it was described that the alternative oxidase is needed for pathogenicity. Here, we investigate expression and function of the alternative oxidase at different stages of the life cycle of the corn pathogen *Ustilago maydis* (Aox1). Interestingly, expression of Aox1 is specifically induced during the stationary phase suggesting a role at high cell density when nutrients become limiting. Studying deletion strains as well as overexpressing strains revealed that Aox1 is dispensable for normal growth, for cell morphology, for response to temperature stress as well as for filamentous growth and plant pathogenicity. However, during conditions eliciting respiratory stress yeast-like growth as well as hyphal growth is strongly affected. We conclude that Aox1 is dispensable for the normal biology of the fungus but specifically needed to cope with respiratory stress.

## Introduction

The alternative oxidase (AOX) is a widespread monotopic protein associated with mitochondria of plants [1, 2], some metazoa (Annelida, Sipuncula, Mollusca, Arthropoda) [3], fungi [1, 4–9] and protists [2, 10–13]. Early structural modeling suggested that the protein contains two hydrophobic regions and two iron binding motifs, which are involved in the transfer of electrons from two molecules of ubiquinol (QH_2_) to oxygen, resulting in the production of water [10]. This architecture was confirmed by the crystal structure of the protein from *Trypanosoma brucei* [14].

AOX facilitates respiratory activity of mitochondria and therefore the functioning of the Krebs cycle in the presence of inhibitors of the cytochrome pathway, such as nitric oxide or cyanide (both inhibitors of complex IV), and antimycin A (inhibitor of complex III) [2, 10, 15]. Furthermore, AOX is specifically inhibited by n-propylgallate (nPG) or n-octylgallate (nOG), and salicylhydroxamic acid (SHAM) [16]. As expected from its monotopic nature, the enzyme does not generate a proton electrochemical potential gradient, and thus it is not involved directly in the synthesis of ATP and the conservation of energy [2]. Regulation of AOX activity is similar in fungi and protists, but different in plants. In plants the enzyme is a homodimer which is activated by the reduction of a disulfide bridge between two cysteine residues located in different subunits of the homodimer, followed by the formation of a thiohemiacetal with a ketoacid, mostly pyruvate [15, 17–22]. In contrast, the AOX in fungi and protists is activated by AMP and GMP [23–26].

So far, the role of the alternative oxidase (AOX) in the majority of the organisms is unclear, but at least four general functions have been proposed for this protein. First, the presence of AOX prevents the inhibition of the Krebs cycle when the mitochondrial cytochrome pathway is blocked [27]. This allows anaplerotic reactions to feed biosynthetic pathways that maintain cell growth and survival under harmful conditions [28]. Second, a thermogenic effect generated by the activity of AOX in some plants has been described [29]. Third, AOX activity is an important cellular mechanism for the prevention of oxidative stress. Indeed, regulation of AOX expression by ROS has been reported for plants [30] and fungal cells [31]. Finally, AOX is important for the development of the parasite *T*. *brucei* [10]. Because the amount of mitochondrial cytochromes is insufficient when *T*. *brucei* is living in the bloodstream of its host, the activity of AOX is essential for the survival of the parasite [10]. Therefore, this enzyme has been proposed as a target for chemotherapeutic treatment of *T*. *brucei* infections [32].

In fungi it has been proposed that AOX participates in the response of cells against different types of stress. This is mainly based on observations that the amount of AOX transcripts is increased during e.g. heat shock in *Aspergillus niger* and *Yarrowia lipolytica* [31, 33], during an oxidative stress in *Aspergillus fumigatus* [34], *A*. *niger* [31], *Magnaporthe grisea* [8], *Candida albicans* [35], *Hansenula anomala* [36], *Paracoccidioides brasiliensis* [37], and under osmotic stress in *A*. *niger* [31]. In pathogenic fungi such as *Cryptococcus neoformans* [38] and *P*. *brasiliensis* [39] virulence was decreased when AOX gene was deleted, suggesting an important role for this enzyme during the infection process.

We study mitochondrial functions in *Ustilago maydis*, a dimorphic fungus of the basidiomycetes phylum, which infects the economically important crop maize [40, 41]. *U*. *maydis* represents a versatile eukaryotic model organism due to its easy cultivation and a set of established molecular, cell biological as well as biochemical tools. In the past *U*. *maydis* has been used to study several biological processes, such as host-parasite relationships [42–45], yeast-mycelium transition [45, 46], gene regulation [47], and more recently, intermediary metabolism [5, 23, 48].

Importantly, in comparison with other model yeasts such as *Saccharomyces cerevisiae*, mitochondria of *U*. *maydis* do contain an electron transport chain with the four classic respiratory complexes (complex I, II, III, and IV), the glycerol 3 phosphate shuttle, and a pair of alternative elements, at least an external NADH dehydrogenase, and the alternative oxidase, Aox1, which is activated by AMP [4, 23]. In contrast with the yeast *S*. *cerevisiae*, which can produce ethanol through fermentation [49], *U*. *maydis* is a fully respiratory microorganism that depends on mitochondrial activity for the synthesis of ATP [5, 50]. Previously, we have shown that inhibition of Aox1 activity by nOG induces lipid peroxidation [51]. In the present study we analyzed the biological function of the enzyme in more detail by combining expression studies and reverse genetics.

## Materials and methods

### Materials

Analytical grade reagents were purchased from Sigma Chemical Co. (St. Louis, MO, USA), E. Merck (Darmstadt, Germany), BioRad (Hercules, CA, USA), Agilent Technologies (La Jolla, CA, USA), Axygen Biosciences (Union City CA, USA), Qiagen (Hilden, Germany), Millipore (Billerica, MA, USA) and Invitrogen/Life Technologies (Darmstadt, Germany). *U*. *maydis* ATCC 201384 FB2 was obtained from the American Type Cell Collection (Manassas, VA, USA).

### Strains and cell cultures

Strains were constructed as described elsewhere [52]. *U*. *maydis* strains were grown at 28°C in rich YPD medium (1.0% glucose, 0.25% peptone, and 0.5% yeast extract), minimal medium (MM) with different carbon sources (1.0% glucose, 0.4% ethanol, 1.0% glycerol or 1.0% lactate) and nitrogen sources (0.3% of ammonium as (NH_4_)_2_SO_4_ or 0.3% of nitrate as KNO_3_), 1x salt and 1x mineral solutions [53]. In all cases, cells were cultured in 100 mL of YPD for 18–24 h, harvested by centrifugation at 1000 g, washed twice with H_2_O, and the final suspension (1 mL/g wet weight) was used to inoculate 1 L of medium with 20 absorption units (A600nm). The cells were harvested at the exponential or stationary phases and suspended with distilled H_2_O at a final ratio of 1 mL/g wet weight. Duplication times were obtained from measurements of the optical density at 600 nm.

### Plasmid construction

*Escherichia coli* Top10 (Invitrogen/Life Technologies) was used for cloning purposes with conventional culture and transformation techniques [54]. Plasmid pUMa2163 was generated using the Golden Gate cloning technology [55]. Briefly, upstream flank (UF) and downstream flank (DF) of *aox1* (accession number umag_02774) were generated by PCR on UM521 genomic DNA using oligonucleotide combinations oRL1400/oRL1401 and oRL1402/oRL1403 (S1 File), respectively. PCR products and pUMa1507 (storage vector I for simple knockout with HygR) were cut and ligated into pUMa1467 (destination vector) in a one-pot BsaI restriction/ligation reaction. For the construction of pUMa2169 a new UF containing the *aox1* ORF and 1015 bps upstream region was generated by PCR using oligonucleotide combination oRL1400/oRL1425 (S1 File) on UM521 genomic DNA. The product was cut with SfiI/XcmI and ligated with a 4300 bps SfiI/XcmI fragment derived from pUMa2163 as well as a 2448 bps SfiI/SfiI fragment derived from pUMa389, containing eGfp and a Nourseothricin-resistance cassette for C-terminal protein fusions [56]. Plasmid constructions were verified by analytical restriction reactions as well as by sequencing of all regions amplified by PCR. For ectopic expression of *aox1*-Gfp under control of the Potef promoter, the ORF of *aox1* was amplified by PCR using oligonucleotide combinations oDD808/oMF894 (with 5 'UTR), and oDD809/oMF894 on UM521 DNA and was introduced into p123 [57].

### Oxygen consumption measurement

Oxygen consumption was measured in a 1.5 mL chamber at 30°C, using a Clark-type electrode connected to an YSI5300A biologic oxygen monitor [4, 23]. The assays were carried out in 20 mM Tris-HCl pH 7.0, 5 to 10 mg of cells (wet weight), and 7.0 mM glucose as substrate. 1.0 mM potassium cyanide (CN) was used to inhibit the cytochrome pathway and 6.0 μM of n-octylgallate (nOG) to inhibit the alternative oxidase (Aox1). Ethanol or dimethyl sulfoxide (DMSO), were used to prepare the 4.0 mM stock solution of nOG.

### Mitochondria isolation

*U*. *maydis* cells were harvested by centrifugation (3000 g, 5 min), washed twice with distilled H_2_O, and resuspended in lysis buffer (300 mM mannitol, 50 mM KH_2_PO_4_, 5.0 mM MgCl_2_, 1.0mM EDTA, 20 mM Hepes-KOH, pH 7.0) to a final ratio of 3.5 mL/g wet weight. Subsequent steps were carried out in the same buffer at 4°C. Cells were disrupted with glass beads, in the presence of 1.0 mM of phenylmethanesulfonyl fluoride (PMSF); the tubes were agitated 4 times for 30 s, at 2 min intervals in a Mini-Beadbeater (Biospec products, USA). Mitochondria were isolated by differential centrifugation as previously described [23]. Briefly, cell debris was eliminated by centrifugation at 3000 g for 10 min. The mitochondrial pellet was obtained by spinning the 3000 g supernatant for 10 min at 12000 g, washed once to eliminate cytosolic contamination, and suspended with lysis buffer to a final protein concentration of 10–30 mg/mL.

### Determination of protein concentration

Protein concentration was determined as described by Lowry et al. [58]. Bovine serum albumin (BSA) was used as standard.

### Microscopy, image quantitative analysis and flow cytometry

Microscopic analysis of sporidial and hyphal cells of *U*. *maydis* was performed as described before [52]. For staining with Mitotracker Red *U*. *maydis* cultures were grown to an OD_600_ = 0,5 incubated with 1 mg/ml Mitotracker Red CM-H_2_ROS (Thermo Fisher, Waltham, MA, USA) for 5 minutes, washed with CM and then directly analysed. For biotin staining of the cell wall, *U*. *maydis* cells were harvested and washed twice with 50 mM PBS (pH 8.0), resuspended in PBS with 1mg/ml biotin and incubated for 30 min at room temperature. Washed thrice with 6x Vol.TM buffer (50 mM Tris-HCl, 50 mM MgCl_2_, pH 7.5), and 1x with 6x Vol. PBS. For subsequent biotin staining with avidin cells were washed twice with 6x Vol. H_2_O, resuspended in 1ml H_2_O + 1 μl Extravidin-TRITC (a modified avidin conjugated to the fluorochrome tetramethyl-rhodamine isothiocyanate; Sigma-Aldrich) and incubated for 10 min at room temperature. Cells were washed twice with 1 ml H_2_O and resuspended in fresh medium. For Aox1-Gfp expression analysis by flow cytometry the Uma1333 (Aox1-Gfp) strain was used. Samples of cells grown in minimal media supplied with ethanol as carbon source, harvested in the stationary phase, incubated in the presence of glucose 1.0% and in the presence or absence of antimycin A were taken every hour for 5 hours. Cellular samples were fixed with paraformaldehyde 0.5% in PBS buffer (monobasic potassium phosphate 3.0 mM; dibasic sodium phosphate 10 mM; sodium chloride 155 mM, pH 7.4) and then the data acquired on a BD Bioscience (Franklin Lakes, NJ, USA), FACScalibur flow cytometer and analysed with the Flow Jo software [59].

### Desiccation test

The desiccation test was adapted from Capece *et al*. 2016. [60]. Cultures of *U*. *maydis* strains were grown until they reached stationary phase. Cultures were then diluted to an OD_600_ of 1.0. Subsequently, 1 ml aliquots of the cell cultures were transferred to 1.5 ml reagent tubes. After centrifugation, the supernatant was discarded and the cell pellets were dried for 0 or 4 hours at 28°C. Subsequently, the pellets were suspended in 1 ml H_2_O and cells resuspended by incubating at 28°C and 1.000 rpm. The resuspended cells were plated on CM + glucose plates and after three days of growth, the colony forming units (cfu) were quantified.

### SDS-PAGE and Aox1 immunodetection

Proteins from mitochondrial preparations were separated by SDS-PAGE on 7.5% (w/v) polyacrylamide slab gels [61], and transferred to a PVDF membrane by a discontinuous buffer system [62]. Nonspecific antibody-binding sites were blocked in a Tris-buffered saline-Tween solution (TBST) with 5% of nonfat milk. Detection of the wild type Aox1 protein was carried out with a monoclonal anti-AOX antibody (dilution 1:100), raised against *Sauromatum guttatum* (voodoo lily) AOX [63]. Detection of Gfp fusion proteins was carried out with a mixture of two monoclonal antibodies against Gfp (clones 7.1 and 13.1, Roche Diagnostics, Basel, Switzerland). Bound antibodies were detected with the appropriate HRP-labeled secondary antibodies and the immobilon western chemiluminescence HRP substrate (Merck Millipore, Darmstadt, Germany).

## Results and discussion

### Aox1 capacity and protein amount is highly elevated during stationary phase

In order to learn more about the function of Aox1 we settled out to investigate enzyme capacity during the saprophytic yeast phase at different nutritional conditions. To this end we tested different nutrients and generated growth curves to define the exponential and stationary phases for each condition. An association between AOX expression and both the growth phase and the carbon source has been observed in other fungi, although the expression patterns depended on the fungus species. For instance, AOX capacity was insignificant during the exponential phase when *Pichia membranifaciens*, *Debaryomyces hansenii*, and *Y*. *lipolytica* were cultured in the presence of glucose, and increased when cells reached the stationary phase [50, 64, 65]. *C*. *albicans* contains two alternative oxidases, Aox1A and Aox1B; Aox1A is constitutive, while Aox1B is expressed during the exponential phase when cells were grown in the presence of non-fermentable sources, but there was no expression of Aox1B in the presence of glucose [66, 67]. When *Pichia pastoris* was cultured in a medium containing glucose as carbon source, the expression of the AOX occurred in both the exponential and stationary phases ([68]).

Glucose, regardless the nitrogen source, and ethanol were good substrates for *U*. *maydis*, growing with duplication times between 2.0 ± 0.1 and 4.9 ± 0.3 h (S1 Fig). For glucose and ethanol 10 h corresponded to the exponential phase and 24 h to the stationary phase (S1 Fig). In contrast, cell growth in the presence of glycerol or lactate was quite slow (S1 Fig), such that 50 h corresponded to the exponential phase, and 125 h to the early stationary phase. Importantly, we can now differentiate precisely between exponential and stationary phases under the different growth conditions.

**Fig 1:**
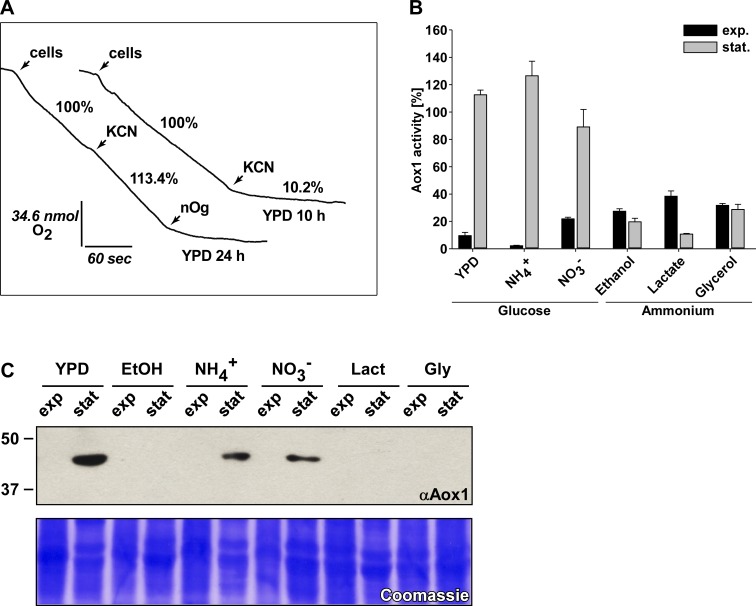
Aox1 is expressed in the stationary phase. **A)** Representative oxygen consumption measurements of FB2 sporidia in exponential and stationary phases. Respiration resistant to potassium cyanide (KCN) in *U*. *maydis* was due to the alternative oxidase (Aox1), which was inhibited by the addition of 6.0 μM of n-octylgallate (nOG). **B)** Percent of Aox1 capacity of cells cultured in several carbon and nitrogen sources and harvested at the exponential and stationary phases. The respiratory activity was determined from the slope obtained before and after the addition of cyanide, as shown in Fig 1A. **C)** Western blot of the Aox1 in mitochondria of cells cultured in different carbon and nitrogen sources. The Coomassie stain shows similar protein load for the different lanes.

Previously, we reported that mitochondrial oxygen consumption by *U*. *maydis* depends on the classic cytochrome pathway (complexes I, II, III, and IV) and the Aox1 [4], which is inhibited by nOG and activated by AMP [23]. Taking advantage of this specific inhibition, we determined the respiratory activity of *U*. *maydis* cells cultured in media with different carbon and nitrogen sources, and harvested in the exponential or stationary phase. As shown in Fig 1A, two types of results were observed upon the addition of cyanide, which can be explained by the amount of Aox1 in the inner mitochondrial membrane. Cells grown in YPD and harvested in the stationary phase showed a more a less continuous rate of oxygen consumption when the cytochrome pathway was inhibited by cyanide, indicating a high capacity of the Aox1. The same holds true for cells cultivated in minimal medium with glucose and ammonium or nitrate as nitrogen sources (Fig 1B). This result was not unexpected since an increase of oxygen uptake in *U*. *maydis* cells upon the addition of antimycin A was described previously [69, 70]. However, for cells grown in the presence of glucose and harvested in the exponential phase, there was a large inhibition of the respiratory activity (80–98%), pointing to a low capacity of the Aox1 (Fig 1A). This effect was observed with the three nitrogen sources and in the presence of glucose (Fig 1B), suggesting that this behavior strongly depends on the growth phase of the cells. For the other carbon sources (ethanol, glycerol, and lactate), an inhibition of the respiratory activity by cyanide was observed in both growth phases (Fig 1B). In all cases, the residual respiratory activity was inhibited by nOG, indicating the participation of Aox1. In essence, higher Aox1 capacities were obtained when cells were grown in the presence of glucose and harvested at stationary phase (Fig 1B). It should be noted that addition of nOG instead of cyanide produced only 0–15% inhibition of oxygen consumption in nearly all conditions, even in the mutant lacking Aox1 (data not shown).

To verify these results we followed two different strategies. Firstly, we detect the amount of Aox1 in mitochondrial protein fractions. To this end we performed Western blot experiments using a monoclonal antibody raised against plant AOX from *S*. *guttatum* (voodoo lily) [23, 63]. The antibody detected only a single band in the expected size of 49 kDa (Fig 1C). In agreement with the results shown in Fig 1B, the presence of Aox1 was observed only in mitochondria isolated from cells grown in the presence of glucose and harvested at the stationary phase (Fig 1C). In contrast, Aox1 was not detected in cells grown in the presence of glucose but harvested in the exponential phase (Fig 1C), or when cells were cultured in ethanol, glycerol or lactate, independent of the growth phase (Fig 1C).

**Fig 2:**
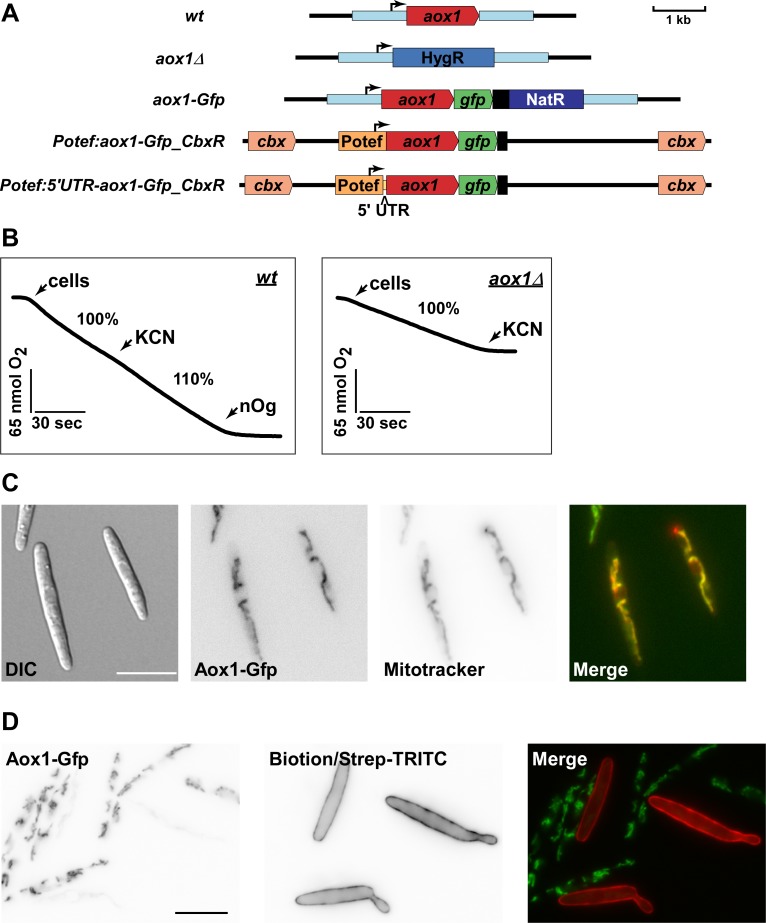
Validation of the function and localization of Aox1-Gfp and other mutants. **A)** Schematic depiction of the genomic loci of the investigated strains. For deletion the ORF of *aox1* was replaced by a hygromycin resistance cassette. The strains with the genotype *aox1-Gfp* express a C-terminal Gfp fusion of *aox1* under control of the native *aox1* promoter. Ectopic expression of aox1-gfp was achieved by introducing a C-terminal fusion with Gfp in the defined *cbx* locus. These constructs were expressed by the constitutive active P_otef_ promoter. Where indicated, constructs included the 65 bp native 5' UTR of *aox1*. **B)** Representative respiration profiles of FB2 (*wt*) and *aox1Δ* sporidia in the stationary phase upon addition of respiratory inhibitors. Potassium cyanide (KCN), inhibits complex IV of the respiratory chain. Residual respiration is due to the activity of alternative oxidase (Aox1) and can be inhibited by addition of n-octylgallate (nOG). Note that FB2aox1Δ does not show any respiration after addition of CN. Furthermore, oxidase capacity of *aox1-Gfp* in FB2aox1-Gfp seems to be attenuated, due to lower oxygen consumption (S2 Fig). **C)** Localization of Aox1-Gfp into the mitochondria of stationary phase sporidia of *U*. *maydis*. Sporidial cells were stained with Mitotracker Red and Aox1-Gfp. Fluorescence signals were analyzed by fluorescence microscopy. **D)** Aox1-Gfp signal can only be seen in the stationary phase of growth. Mixed culture of sporidial cells in exponential phase (OD600 ~0.5) labeled with biotin/avidin-TRITC, and a stationary phase culture of *U*. *maydis*.

Secondly, we generated strains expressing Aox1 fused at its C-terminus with Gfp (Aox1-Gfp) at the homologous locus of laboratory strain FB2. In order to test the functionality of the fusion protein we compared its capacity to wild type and to gene deletion mutant *aox1Δ* (Fig 2A). Testing the latter strains revealed that, as expected, no alternative oxidase activity could be measured (Fig 2B). Aox1-Gfp was clearly functional although its capacity was reduced in comparison to wild type (Fig 2B).

Studying cells grown to stationary phase using fluorescence microscopy revealed that Aox1-Gfp localized to mitochondria *in vivo* (Fig 2C). In order to compare the amount of Aox1-Gfp in mitochondria from exponentially grown cells and cells from the stationary phase we analyzed a mixture of cells simultaneously (Fig 2D, exponentially grown cells were labeled with biotin/avidin-TRITC for identification). This showed unambiguously that mitochondrial Aox1-Gfp was only detected in cells from the stationary phase. In summary, Aox1 capacity and expression was specifically induced when cells enter the stationary phase.

At first sight, this behavior makes sense. In the exponential phase, cells are rapidly dividing and engaged in biosynthetic processes supported by ATP [65, 71]. Since Aox1 decreases the efficiency of the oxidative phosphorylation its activity should be turned off or decreased when cells have a high requirement of ATP. However, cells in the stationary phase will face an environment with hypoxia, due to the high cell density reached at the end of the exponential phase and the large respiratory capacity of *U*. *maydis* cells. These are indeed the conditions for reduction of the ubiquinone pool in mitochondria and the generation of reactive oxygen species (ROS). Since AOX has been implicated in the protection against ROS, the increase in AOX capacity at the stationary phase might be related to this specific function. However, with ethanol, lactate, and glycerol the Aox1 capacity was approximately the same when cells moved from the exponential into the stationary phase (Fig 1B) pointing to a specific effect of glucose on the expression and/or capacity of Aox1 during the growth curve. In agreement with our results AOX was present in both phases when *D*. *hansenii* was grown with lactate as the carbon source, suggesting that this effect is related with the presence of glucose and the growth phase [65].

### Aox1 expression is transcriptionally induced at the early stationary phase

**Fig 3:**
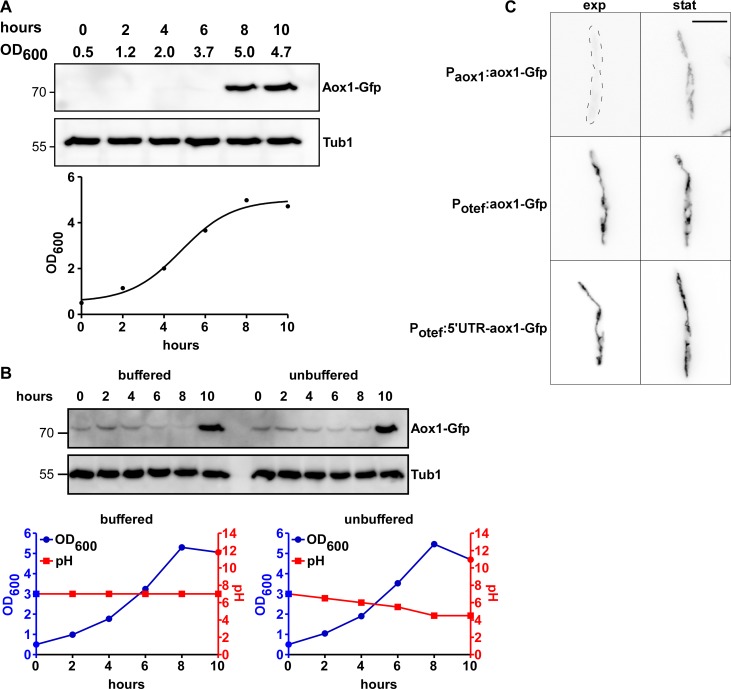
Expression of Aox1-Gfp. **A)** Expression of Aox1-Gfp dependent on OD and growth phase. Aox1-Gfp is expressed in the stationary phase. Aox1-Gfp was detected by anti-Gfp. Tub1 serves as loading control. **B)** Expression of Aox1-Gfp is not dependent on acidification of the media. Western Blot, growth curve, and pH values of the media (unbuffered = CM; buffered = CM + 100 mM MOPS) depending on time. **C)** Sporidia of FB2, FB2Potef:aox1-Gfp, and FB2Potef:5'UTR-aox1-Gfp showing that aox1-Gfp under the control of the otef-promoter is expressed irrespective of the growth phase.

In order to analyze the precise timing of Aox1 expression we performed time course experiments with Aox1-Gfp. We observed an increase of Aox1-Gfp amount at the onset of the stationary phase (Fig 3A). Since changes in pH of the culture medium during the cell growth might be associated with the expression of Aox1 we grew the cells in the presence of 100 mM MOPS (pH 7) to avoid acidification. Changes in the amount of Aox1 during incubation time were detected in cell extracts using anti-Gfp antibodies. The expression pattern of Aox1 was the same regardless of the acidification of the extracellular environment (Fig 3B). This observation is in agreement with our results that the pH value of the cultivation medium did not have any influence on the growth of FB2 or FB2aox1Δ (S3 Fig).

To explore the main regulatory step of Aox1 expression we constructed two mutants in which the wild type promoter was replaced by the constitutively active promoter P_otef_ (Fig 2A). In one case we included the 5´ untranslated region of *aox1* to account for potential translational regulation (P_otef_:5´UTR-aox1-Gfp; Fig 2A). In both cases Aox1 capacity was comparable to wild type (S2 Fig), indicating that the increased expression of Aox1-Gfp rescued the slight defect of the C-terminal fusion of Aox1 with Gfp (compare Fig 2B and S2 Fig) [72].

Analyzing these strains revealed that the Aox1-Gfp amount was detectable at the same levels during the exponential and stationary phases (Fig 3C). This suggests that the main regulatory step takes place at the transcriptional level since the constitutive synthesis of *aox1* mRNA resulted in the presence of the Aox1-Gfp in mitochondria from cells harvested in both the exponential and stationary growth phases. In some plants and fungi, the presence of oxidative stress [73, 74], low or high temperature [75, 76], or osmotic stress [77–79], induces an increase in the concentration of AOX in mitochondria [66]. In the majority of the cases, this was associated with an activation of the synthesis of RNA, indicating that the expression of AOX in plants and fungi is mainly regulated at the transcriptional level [80–82].

### Aox1 is dispensable for yeast-like growth and for the response to different temperatures

**Fig 4:**
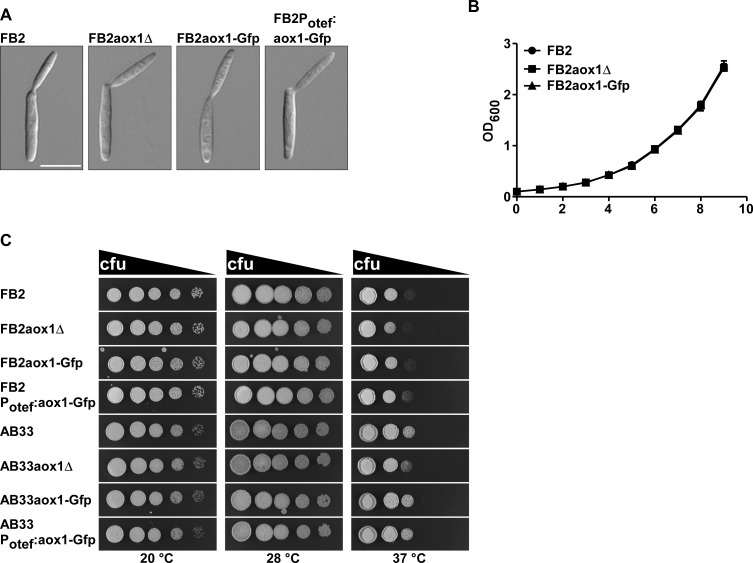
Effect of Aox1 on the sporidia growth of *U*. *maydis* and sensitivity to temperature. **A)** Budding of yeast cells. Sporidia of FB2 (wt), FB2aox1Δ, FB2aox1-Gfp, and FB2Potef:aox1-Gfp. **B)** Time course of cell growth in rich medium as measured by the absorbance at 600 nm. **C)** Effect of temperature on the growth of sporidia. Growth plates with 1:5 dilutions (starting with OD = 0.5).

Next we wanted to learn more about the biological role of Aox1. It is difficult to interpret the results from experiments with the AOX inhibitors because they affect other processes in cells. For example, gallates inhibit the growth of *S*. *cerevisiae* in spite of the absence of an AOX in this organism [83, 84]. Therefore, we used the *aox1*Δ mutant (Fig 2A) to study the participation of this enzyme at different stages of the life cycle of *U*. *maydis*. First we used the FB2 strain to analyze the influence of Aox1 on the saprophytic growth of *U*. *maydis* [57]. In contrast to other reports in fungi (see below) we did not observe any difference between the FB2 wild type and the *aox1*Δ mutant. Cell budding (Fig 4A), growth rate in complete medium (Fig 4B), and sensitivity to low (20°C) or high (37°C) temperatures (Fig 4C) were basically the same regardless the presence of Aox1, indicating that this enzyme was not involved in any of these processes. The growth rate of FB2 and FB2aox1Δ was even comparable when using different carbon sources (S3 Fig). It is worth noting that similar growth rates for wild type and the mutant lacking Aox1 cultured in liquid rich medium were reported previously in *U*. *maydis* [69].

**Fig 5:**
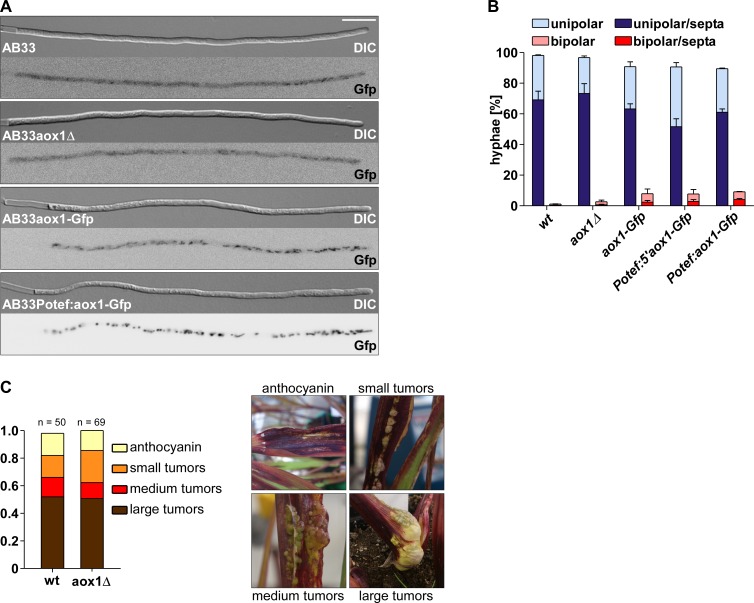
Effect of Aox1 on the growth of *U*. *maydis* and infectivity. **A)** Filamentous growth of *U*. *maydis* AB33 derivatives 8 h.p,i., size bar, 10 μm. DIC and Gfp fluorescence images are depicted for each strain. Note that the Gfp-labelled images in AB33 and AB33aox1Δ depict unspecific fluorescence, which is detectable with low intensity in these control strains. **B)** Percentage of hyphae (8 h.p.i.): unipolarity, bipolarity, and septum formation was quantified (error bars, s.e.m.; n = 3 independent experiments; >100 hyphae were counted per experiment; note that septum formation is given relative to the values of unipolar or bipolar hyphae set to 100%). **C)** Results of plant infection experiments with wt strains (FB1 x FB2) and the *aox1Δ* strains (FB1aox1Δ x FB2aox1Δ). The percentage of plants with typical disease symptoms is given (two experiments, at least 50 plants infected with each strain).

Next we studied both the filamentous growth using the AB33 strain (wt and *aox1Δ*) and corn infection using the compatible FB1 and FB2 strains. Deletion of the *aox1* gene had no effect on the filamentous growth of the cell as hyphae grew unipolarly, and inserted septa at the basal pole (Fig 5A). Also the rate of bipolar cells were not drastically increased (Fig 5B) suggesting that microtubule-dependent processes were not disturbed, since it is known that this results in an increased rate of bipolarity [52, 85]. Also, overexpression of the enzyme did hardly affect the filamentous growth programme although we observed a slight increase in the amount of bipolar hyphae in strains expressing Aox1-Gfp (Fig 5A and 5B). More interestingly, infection of the maize plant was not affected by the absence of the Aox1 (Fig 5C) indicating that this enzyme does not have a role during the initial infection and the further development of *U*. *maydis* inside the plant.

In several pathogenic fungi, this enzyme participates in cellular growth, morphological transitions (yeast-mycelium) [37, 86], and in some cases in the infection of the specific host [87, 88]. Incubation of *Sclerotinia sclerotiorum* in the presence of salicylhydroxamic acid (SHAM) inhibits the mycelial growth of the organism and diminished the biomass yield [89]. Similar results were obtained with *Nomuraea rileyi* [90]. Either incubation of *N*. *rileyi* with SHAM or knock-down of the AOX gene drastically changed the hyphae morphology, decreased the microsclerotial production, and reduced the growth yield of the fungus [90]. Mutants of *C*. *neoformans*, *P*. *brasiliensis*, and *C*. *albicans* lacking the AOX showed low virulence and low tolerance to oxidative and other types of stress [37, 38, 66, 91]. In *M*. *grisea* loss of Aox1 had no effect on the pathogenicity and virulence of this organism during the infection of barley leaves. Interestingly, AOX was essential when the infection assays were conducted in the presence of azoxystrobin, a fungicide that interacts with the respiratory complex III of *M*. *grisea* [92].

### Aox1 is crucial in coping with respiratory stress in the yeast and hyphal form

**Fig 6:**
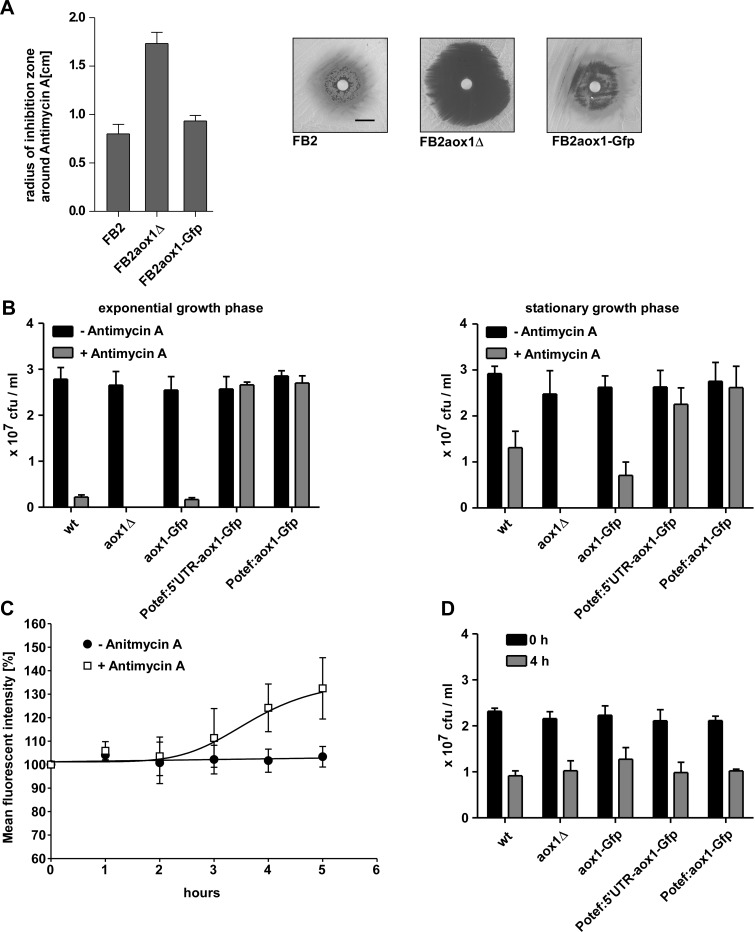
Effect of the presence of Aox1 on the sensitivity of sporidia to antimycin A. **A)** Sensitivity of *U*. *maydis* WT (FB2) and mutants (FB2aox1Δ and FB2aox1-Gfp) to antimycin A. 10 μl of antimycin A (10 mg/ml) was placed in the center of the plate with *U*. *maydis* cells, and the radius of inhibition of the growth for FB2, FB2aox1Δ and FB2aox1-Gfp was measured. **B)** Colony forming units obtained in presence or absence of antimycin A by cells harvested at the exponential and stationary phases. **C)** Induction of Aox1-Gfp expression by antimycin A (2 μM final concentration). The fluorescent signal was measured by flow cytometry. **D)** Sensitivity of *U*. *maydis* WT and mutants to dessication.

Thus far we only tested normal unstressed conditions. In order to investigate a role of Aox1 during respiratory stress we used antimycin A to block complex III resulting in respiratory defects. Like some commercial antifungal molecules that inhibit complex III (azoxystrobin or metominostrobin [93]), antimicyn A also induces a respiratory stress. Loss of Aox1 renders yeast-like cells of *U*. *maydis* completely sensitive to antimycin A whereas the growth of the wild type and of strains expressing Aox1-Gfp was similar, supporting our previous conclusion that Aox1-Gfp was at least partially functional. To better outline the differences between cells lacking Aox1 (FB2aox1Δ), cells containing normal amounts of Aox1 but with different activities (WT, FB2aox1-Gfp), and cells overexpressing the enzyme (P_otef_:5’UTR-aox1-Gfp and P_otef_:aox1-Gfp) we measured the number of colonies forming units (cfu) in the presence or absence of antimycin A. In the absence of the inhibitor, the number of colonies was independent of the presence of Aox1 or the expression levels of this enzyme in cells from both the exponential and stationary growth phases, indicating similar viability in all conditions (Fig 6B). Growth in the presence of antimycin A was fully inhibited in cells lacking the *aox1* gene, regardless of the growth phase, indicating the importance of this enzyme for cell growth when the cytochrome pathway was inhibited (complex III or IV; Fig 6A and 6B). For cells expressing the normal amount of the protein, FB2 and FB2aox1-Gfp strains, their viability on media containing antimycin A was smaller in the exponential phase than in the stationary phase in agreement with the lower amount of Aox1 in the exponential phase (Fig 6A and 6B). Interestingly, viability or cell survival in the presence of antimycin A was fully recovered when cells overexpressed the Aox1 (P_otef_:5’UTR-aox1-Gfp and P_otef_: aox1-Gfp), in both growth phases (Fig 6B), indicating that the alternative oxidase is important to deal with the inhibition of the respiratory chain, allowing the generation of a proton motive force via complex I, and restoring the growth capacity. In agreement with previous reports the presence of antimycin A induced the synthesis of Aox1 (Fig 6C) [23], pointing to an unknown regulatory circuit. It has been proposed that the signal underlying the synthesis of AOX is an increase in ROS, specifically hydrogen peroxide [73, 74]. A recent study reports that the AOX was essential to maintain mitochondrial respiration when plants were exposed to drought, an abiotic stress that inhibits the cytochrome pathway [94]. In order to test for a similar function of Aox1 in *U*. *maydis*, we subjected FB2 derivates to desiccation. However, comparable survival rates were obtained for all *aox1* alleles (Fig 6D).

**Fig 7:**
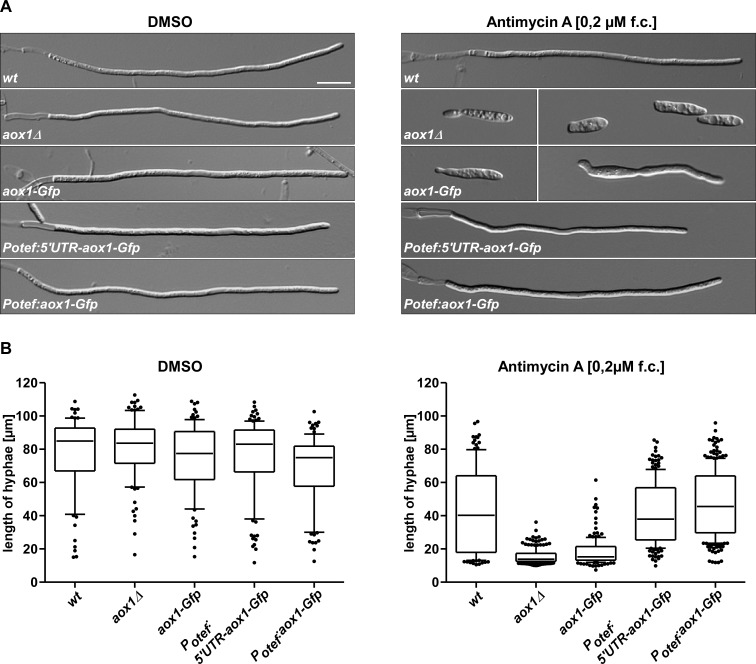
Effect of the presence of Aox1 and antimycin A on the filamentous growth of *U*. *maydis*. **A)** Filamentous growth of *U*. *may*dis AB33 wt and mutants in the presence or absence of antimycin A. **B)** Measurement of the length of the hyphae formed by *U*. *maydis* AB33 and derivatives in the presence or absence of antimycin A.

Importantly, the same protection of Aox1 during respiratory stress is also operational during hyphal growth. In the absence of antimycin A growth of AB33 strains (*wt*, *aox1Δ*, *aox1-Gfp*, *P*_*otef*_:*5’UTR-aox1-Gfp* and *P*_*otef*_:*aox1-Gfp*), measured by the increase in the length of the hyphae, was basically the same independent of the expression of Aox1 (Fig 7A and 7B). With antimycin A in the medium only the wt (AB33) and the two strains overexpressing the protein (AB33P_otef_:5’UTR-aox1-Gfp, and AB33P_otef_:aox1-Gfp) displayed normal filamentous growth. In the aox1Δ, filamentous growth was fully inhibited, whereas cells containing the fusion of Aox1 with Gfp showed a reduced filamentous growth, suggesting that the smaller capacity of Aox1-Gfp was not enough to support the synthesis of ATP required for this process. In summary, in both growth forms of *U*. *maydis*, i.e. yeast-like and hyphal growth, Aox1 is crucial to cope with respiratory stress. With few exceptions plants and fungi increase their AOX capacity when incubated with H_2_O_2_, menadione or paraquat [34, 66, 67], suggesting an important role of the AOX to cope with oxidative stress. Western blot analysis also indicates an increase in the mitochondrial AOX content for some of these studies [66] and also an increase in AOX transcripts [81, 82]. After a 3–4 h incubation of *U*. *maydis* sporidia in the presence of sodium azide, cyanide or antimycin A, there was an increase in the rate of oxygen uptake in the presence of cyanide [69, 70] pointing to an increase in the AOX content.

## Conclusion

Aox1 is specifically induced during the stationary phase when cells are cultured in the presence of glucose, independent of the nitrogen source. With other carbon sources the expression of Aox1 was much smaller than with glucose and approximately the same in both growth phases. Acidification of the growth media was not implicated in the expression of Aox1 in the stationary phase. It seems that in rich YPD medium expression of Aox1 is mainly regulated at the transcriptional level. In spite of the many roles proposed for AOX in fungal cells, in *U*. *maydis* this enzyme does not participate in the growth of yeast cells or in the adaptation of cells to high or low temperatures. Aox1 is dispensable for infectious growth and plant infection. However, Aox1 allows the cells to grow in the presence of respiratory chain inhibitors. Thus, although Aox1 is not essential for the known normal biology of the fungus, it is clearly needed to cope with respiratory stress at different stages of the life cycle. It is important to note that Aox1 might carry out additional functions during specific environmental conditions that escaped our analysis, such as the survival and pathogenicity of *U*. *maydis* in the presence of fungicides.

## Supporting information

S1 File Description of strains, plasmids and oligonucleotides used in this study. (PDF)

S1 Fig Growth curves of *Ustilago maydis*. *Ustilago maydis* was grown in different carbon and nitrogen sources and the absorbance at 600nm measured at the indicated time. **YPD**, rich medium (1.0% glucose, 0.25% peptone, and 0.5% yeast extract); **NH**_**4**_^**+**^, minimal medium with glucose (1.0%) and ammonium sulfate (0.3%); **NO**_**3**_^**-**^, minimal medium with glucose (1.0%) and potassium nitrate (0.3%); **EtOH**, minimal medium with ethanol (0.4%) and ammonium sulfate (0.3%); **Lactate**, minimal medium with lactate (1.0%) and ammonium sulfate (0.3%); **Glycerol**, minimal medium with glycerol (1.0%) and ammonium sulfate (0.3%).
(TIF)

S2 Fig Respiratory activity of strains with the Aox1 fuse to Gfp. Oxygen consumption was measured as indicated under material and methods. Respiratory traces of ***aox1-Gfp***, ***Potef*:*5’UTR-aox1-Gfp***, and ***Potef*:*aox1-Gfp*** sporidia in the stationary phase. Arrows show the addition of KCN and n-octylgallate (nOG).
(TIF)

S3 Fig Growth curves of *Ustilago maydis* FB2 and FB2aox1Δ. *Ustilago maydis* wild type and the strain lacking *aox1* were grown in different carbon sources as in S1 Fig, using ammonium sulfate (0.3%) as nitrogen source. Minimal medium with glucose was prepared with or without 50 mM MOPS.
(TIF)

## References

[1] Albury MS, Elliott C, Moore AL. Towards a structural elucidation of the alternative oxidase in plants. Physiol Plant. 2009;137(4):316–27. 10.1111/j.1399-3054.2009.01270.x 19719482

[2] Moore AL, Albury MS. Further insights into the structure of the alternative oxidase: from plants to parasites. Biochem Soc Trans. 2008;36(Pt 5):1022–6. 10.1042/BST0361022 18793182

[3] McDonald AE, Vanlerberghe GC, Staples JF. Alternative oxidase in animals: unique characteristics and taxonomic distribution. J Exp Biol. 2009;212(Pt 16):2627–34. 10.1242/jeb.032151 19648408

[4] Juarez O, Guerra G, Martinez F, Pardo JP. The mitochondrial respiratory chain of *Ustilago maydis*. Biochim Biophys Acta. 2004;1658(3):244–51. 10.1016/j.bbabio.2004.06.005 15450962

[5] Juarez O, Guerra G, Velazquez I, Flores-Herrera O, Rivera-Perez RE, Pardo JP. The physiologic role of alternative oxidase in *Ustilago maydis*. Febs J. 2006;273(20):4603–15. 10.1111/j.1742-4658.2006.05463.x 16965537

[6] Veiga A, Arrabaca JD, Loureiro-Dias MC. Cyanide-resistant respiration is frequent, but confined to yeasts incapable of aerobic fermentation. FEMS Microbiol Lett. 2000;190(1):93–7. 10981696 10.1111/j.1574-6968.2000.tb09268.x

[7] Veiga A, Arrabaca JD, Loureiro-Dias MC. Cyanide-resistant respiration, a very frequent metabolic pathway in yeasts. FEMS Yeast Res. 2003;3(3):239–45. 12689632 10.1016/S1567-1356(03)00036-9

[8] Yukioka H, Inagaki S, Tanaka R, Katoh K, Miki N, Mizutani A, et al Transcriptional activation of the alternative oxidase gene of the fungus *Magnaporthe grisea* by a respiratory-inhibiting fungicide and hydrogen peroxide. Biochim Biophys Acta. 1998;1442(2–3):161–9. 9804939 10.1016/s0167-4781(98)00159-6

[9] Lambowitz AM, Sabourin JR, Bertrand H, Nickels R, McIntosh L. Immunological identification of the alternative oxidase of *Neurospora crassa* mitochondria. Mol Cell Biol. 1989;9(3):1362–4. 2524649 10.1128/mcb.9.3.1362-1364.1989 PMC362733

[10] Chaudhuri M, Ott RD, Hill GC. Trypanosome alternative oxidase: from molecule to function. Trends Parasitol. 2006;22(10):484–91. 10.1016/j.pt.2006.08.007 16920028

[11] Jarmuszkiewicz W, Wagner AM, Wagner MJ, Hryniewiecka L. Immunological identification of the alternative oxidase of *Acanthamoeba castellanii* mitochondria. FEBS Lett. 1997;411(1):110–4. 9247153 10.1016/s0014-5793(97)00676-5

[12] Castro-Guerrero NA, Krab K, Moreno-Sanchez R. The alternative respiratory pathway of euglena mitochondria. J Bioenerg Biomembr. 2004;36(5):459–69. 10.1023/B:JOBB.0000047328.82733.ef 15534393

[13] Chaudhuri M, Ott RD, Saha L, Williams S, Hill GC. The trypanosome alternative oxidase exists as a monomer in *Trypanosoma brucei* mitochondria. Parasitol Res. 2005;96(3):178–83. 10.1007/s00436-005-1337-3 15864649

[14] Shiba T, Kido Y, Sakamoto K, Inaoka DK, Tsuge C, Tatsumi R, et al Structure of the trypanosome cyanide-insensitive alternative oxidase. Proc Natl Acad Sci U S A. 2013;110(12):4580–5. 10.1073/pnas.1218386110 23487766 PMC3607012

[15] Moore AL, Shiba T, Young L, Harada S, Kita K, Ito K. Unraveling the heater: new insights into the structure of the alternative oxidase. Annu Rev Plant Biol. 2013;64:637–63. 10.1146/annurev-arplant-042811-105432 23638828

[16] Grahl N, Dinamarco TM, Willger SD, Goldman GH, Cramer RA. *Aspergillus fumigatus* mitochondrial electron transport chain mediates oxidative stress homeostasis, hypoxia responses and fungal pathogenesis. Mol Microbiol. 2012;84(2):383–99. 10.1111/j.1365-2958.2012.08034.x 22443190 PMC3323727

[17] Moore AL, Siedow JN. The regulation and nature of the cyanide-resistant alternative oxidase of plant mitochondria. Biochim Biophys Acta. 1991;1059(2):121–40. 1883834 10.1016/s0005-2728(05)80197-5

[18] Moore AL, Siedow JN. The nature and regulation of the alternative oxidase of plant mitochondria. Biochem Soc Trans. 1992;20(2):361–3. 1397631 10.1042/bst0200361

[19] Day DA, Millar AH, Wiskich JT, Whelan J. Regulation of Alternative Oxidase Activity by Pyruvate in Soybean Mitochondria. Plant Physiol. 1994;106(4):1421–7. 12232419 10.1104/pp.106.4.1421 PMC159681

[20] Millar AH, Wiskich JT, Whelan J, Day DA. Organic acid activation of the alternative oxidase of plant mitochondria. FEBS Lett. 1993;329(3):259–62. 8365467 10.1016/0014-5793(93)80233-k

[21] Affourtit C, Albury MS, Crichton PG, Moore AL. Exploring the molecular nature of alternative oxidase regulation and catalysis. FEBS Lett. 2002;510(3):121–6. 11801238 10.1016/s0014-5793(01)03261-6

[22] McIntosh L. Molecular biology of the alternative oxidase. Plant Physiol. 1994;105(3):781–6. 8058835 10.1104/pp.105.3.781 PMC160724

[23] Sierra-Campos E, Velazquez I, Matuz-Mares D, Villavicencio-Queijeiro A, Pardo JP. Functional properties of the *Ustilago maydis* alternative oxidase under oxidative stress conditions. Mitochondrion. 2009;9(2):96–102. 10.1016/j.mito.2009.01.003 19460302

[24] Joseph-Horne T, Hollomon DW, Wood PM. Fungal respiration: a fusion of standard and alternative components. Biochim Biophys Acta. 2001;1504(2–3):179–95. 11245784 10.1016/s0005-2728(00)00251-6

[25] Jarmuszkiewicz W, Czarna M, Sluse FE. Substrate kinetics of the *Acanthamoeba castellanii* alternative oxidase and the effects of GMP. Biochim Biophys Acta. 2005;1708(1):71–8. 10.1016/j.bbabio.2005.01.003 15949985

[26] Michea-Hamzehpour M, Turian G. GMP-stimulation of the cyanide-insensitive mitochondrial respiration in heat-shocked conidia of *Neurospora crassa*. Experientia. 1987;43(4):439–40. 3032673 10.1007/BF01940445

[27] Hans L. Cyanide-respistant respiration: A non-phosphorylating electron transport pathway acting as an energy overflow. Physiol Plant. 1982;55:478–85.

[28] Moore AL, Rich PR. The bioenergetics of plant mitochondria. Trends Biochem Sci. 1980;5:284–8.

[29] Zhu Y, Lu J, Wang J, Chen F, Leng F, Li H. Regulation of thermogenesis in plants: the interaction of alternative oxidase and plant uncoupling mitochondrial protein. J Integr Plant Biol. 2011;53(1):7–13. 10.1111/j.1744-7909.2010.01004.x 21205176

[30] Clifton R, Lister R, Parker KL, Sappl PG, Elhafez D, Millar AH, et al Stress-induced co-expression of alternative respiratory chain components in *Arabidopsis thaliana*. Plant Mol Biol. 2005;58(2):193–212. 10.1007/s11103-005-5514-7 16027974

[31] Honda Y, Hattori T, Kirimura K. Visual expression analysis of the responses of the alternative oxidase gene (aox1) to heat shock, oxidative, and osmotic stresses in conidia of citric acid-producing *Aspergillus niger*. J Biosci Bioeng. 2012;113(3):338–42. 10.1016/j.jbiosc.2011.10.026 22138384

[32] Nihei C, Fukai Y, Kita K. Trypanosome alternative oxidase as a target of chemotherapy. Biochim Biophys Acta. 2002;1587(2–3):234–9. 12084465 10.1016/s0925-4439(02)00086-8

[33] Biriukova EN, Medentsev AG, Arinbasarova A, Akimenko VK. Respiratory activity of yeast *Yarrowia lipolytica* under oxidative stress and heat shock. Mikrobiologiia. 2008;77(4):448–52. 18825969

[34] Magnani T, Soriani FM, Martins VP, Nascimento AM, Tudella VG, Curti C, et al Cloning and functional expression of the mitochondrial alternative oxidase of *Aspergillus fumigatus* and its induction by oxidative stress. FEMS Microbiol Lett. 2007;271(2):230–8. 10.1111/j.1574-6968.2007.00716.x 17425662

[35] Yan L, Li M, Cao Y, Gao P, Cao Y, Wang Y, et al The alternative oxidase of *Candida albicans* causes reduced fluconazole susceptibility. J Antimicrob Chemother. 2009;64(4):764–73. 10.1093/jac/dkp273 19656781

[36] Minagawa N, Koga S, Nakano M, Sakajo S, Yoshimoto A. Possible involvement of superoxide anion in the induction of cyanide-resistant respiration in *Hansenula anomala*. FEBS Lett. 1992;302(3):217–9. 1318225 10.1016/0014-5793(92)80444-l

[37] Martins VP, Dinamarco TM, Soriani FM, Tudella VG, Oliveira SC, Goldman GH, et al Involvement of an alternative oxidase in oxidative stress and mycelium-to-yeast differentiation in *Paracoccidioides brasiliensis*. Eukaryot Cell. 2011;10(2):237–48. 10.1128/EC.00194-10 21183691 PMC3067407

[38] Akhter S, McDade HC, Gorlach JM, Heinrich G, Cox GM, Perfect JR. Role of alternative oxidase gene in pathogenesis of *Cryptococcus neoformans*. Infect Immun. 2003;71(10):5794–802. 10.1128/IAI.71.10.5794-5802.2003 14500501 PMC201089

[39] Ruiz OH, Gonzalez A, Almeida AJ, Tamayo D, Garcia AM, Restrepo A, et al Alternative oxidase mediates pathogen resistance in *Paracoccidioides brasiliensis* infection. PLoS Negl Trop Dis. 2011;5(10):e1353 10.1371/journal.pntd.0001353 22039556 PMC3201906

[40] Brefort T, Doehlemann G, Mendoza-Mendoza A, Reissmann S, Djamei A, Kahmann R. *Ustilago maydis* as a Pathogen. Annu Rev Phytopathol. 2009;47:423–45. 10.1146/annurev-phyto-080508-081923 19400641

[41] Dean R, Van Kan JA, Pretorius ZA, Hammond-Kosack KE, Di Pietro A, Spanu PD, et al The Top 10 fungal pathogens in molecular plant pathology. Mol Plant Pathol. 2012;13(4):414–30. 10.1111/j.1364-3703.2011.00783.x 22471698 PMC6638784

[42] Djamei A, Kahmann R. *Ustilago maydis*: dissecting the molecular interface between pathogen and plant. PLoS Pathog. 2012;8(11):e1002955 10.1371/journal.ppat.1002955 23133380 PMC3486881

[43] Schirawski J, Mannhaupt G, Munch K, Brefort T, Schipper K, Doehlemann G, et al Pathogenicity determinants in smut fungi revealed by genome comparison. Science. 2010;330(6010):1546–8. 10.1126/science.1195330 21148393

[44] Kahmann R, Kämper J. *Ustilago maydis*: how its biology relates to pathogenic development. New Phytol. 2004(164):31–42. 10.1111/j.1469-8137.2004.01156.x 33873482

[45] Leuthner B, Aichinger C, Oehmen E, Koopmann E, Muller O, Muller P, et al A H_2_O_2_-producing glyoxal oxidase is required for filamentous growth and pathogenicity in *Ustilago maydis*. Mol Genet Genomics. 2005;272(6):639–50. 10.1007/s00438-004-1085-6 15578222

[46] Feldbrugge M, Kamper J, Steinberg G, Kahmann R. Regulation of mating and pathogenic development in *Ustilago maydis*. Curr Opin Microbiol. 2004;7(6):666–72. 10.1016/j.mib.2004.10.006 15556041

[47] Kaffarnik F, Muller P, Leibundgut M, Kahmann R, Feldbrugge M. PKA and MAPK phosphorylation of Prf1 allows promoter discrimination in *Ustilago maydis*. EMBO J. 2003;22(21):5817–26. 10.1093/emboj/cdg554 14592979 PMC275411

[48] Saavedra E, Ramos-Casillas LE, Marin-Hernandez A, Moreno-Sanchez R, Guerra-Sanchez G. Glycolysis in *Ustilago maydis*. FEMS Yeast Res. 2008;8(8):1313–23. 10.1111/j.1567-1364.2008.00437.x 18803552

[49] Pena A, Cinco G, Puyou AG, Tuena M. Studies on the mechanism of the stimulation of glycolysis and respiration by K+ in *Saccharomyces cerevisiae*. Biochim Biophys Acta. 1969;180(1):1–8. 4239611 10.1016/0005-2728(69)90187-x

[50] Guerrero-Castillo S, Cabrera-Orefice A, Vazquez-Acevedo M, Gonzalez-Halphen D, Uribe-Carvajal S. During the stationary growth phase, *Yarrowia lipolytica* prevents the overproduction of reactive oxygen species by activating an uncoupled mitochondrial respiratory pathway. Biochim Biophys Acta. 2012;1817(2):353–62. 10.1016/j.bbabio.2011.11.007 22138628

[51] Sierra-Campos E, Valdez-Solana MA, Matuz-Mares D, Velazquez I, Pardo JP. Induction of morphological changes in *Ustilago maydis* cells by octyl gallate. Microbiology. 2009;155(Pt 2):604–11. 10.1099/mic.0.020800-0 19202109

[52] Pohlmann T, Baumann S, Haag C, Albrecht M, Feldbrugge M. A FYVE zinc finger domain protein specifically links mRNA transport to endosome trafficking. Elife. 2015;4. 10.7554/eLife.06041 PMC4466420 25985087

[53] Holliday R. Ustilago maydis In: King R, editor. Handbook of Genetics. New York: Plenum; 1974 p. 575–95.

[54] Sambrook J FE, Maniatis T. Molecular cloning: a laboratory manual. Cold Spring Harbor, New York: Cold Spring Harbor Laboratory; 1989.

[55] Terfruchte M, Joehnk B, Fajardo-Somera R, Braus GH, Riquelme M, Schipper K, et al Establishing a versatile Golden Gate cloning system for genetic engineering in fungi. Fungal Genet Biol. 2014;62:1–10. 10.1016/j.fgb.2013.10.012 24211735

[56] Brachmann A, Konig J, Julius C, Feldbrugge M. A reverse genetic approach for generating gene replacement mutants in *Ustilago maydis*. Mol Genet Genomics. 2004;272(2):216–26. 10.1007/s00438-004-1047-z 15316769

[57] Brachmann A, Weinzierl G, Kamper J, Kahmann R. Identification of genes in the bW/bE regulatory cascade in *Ustilago maydis*. Mol Microbiol. 2001;42(4):1047–63. 11737646 10.1046/j.1365-2958.2001.02699.x

[58] Lowry OH, Rosebrough NJ, Farr AL, Randall RJ. Protein measurement with the Folin phenol reagent. J Biol Chem. 1951;193(1):265–75. 14907713

[59] Pinon-Zarate G, Herrera-Enriquez MA, Hernandez-Tellez B, Jarquin-Yanez K, Castell-Rodriguez AE. GK-1 improves the immune response induced by bone marrow dendritic cells loaded with MAGE-AX in mice with melanoma. J Immunol Res. 2014;2014:158980 10.1155/2014/158980 25759825 PMC4230216

[60] Capece A, Votta S, Guaragnella N, Zambuto M, Romaniello R, Romano P. Comparative study of *Saccharomyces cerevisiae* wine strains to identify potential marker genes correlated to desiccation stress tolerance. FEMS Yeast Res. 2016;16(3). 10.1093/femsyr/fow015 26882930

[61] Laemmli UK. Cleavage of structural proteins during the assembly of the head of bacteriophage T4. Nature. 1970;227(5259):680–5. 5432063 10.1038/227680a0

[62] Lauriere M. A semidry electroblotting system efficiently transfers both high- and low-molecular-weight proteins separated by SDS-PAGE. Anal Biochem. 1993;212(1):206–11. 10.1006/abio.1993.1313 8368495

[63] Elthon TE, Nickels RL, McIntosh L. Monoclonal antibodies to the alternative oxidase of higher plant mitochondria. Plant Physiol. 1989;89(4):1311–7. 16666702 10.1104/pp.89.4.1311 PMC1056014

[64] Veiga A, Arrabaca JD, Loureiro-Dias MC. Stress situations induce cyanide-resistant respiration in spoilage yeasts. J Appl Microbiol. 2003;95(2):364–71. 12859770 10.1046/j.1365-2672.2003.01992.x

[65] Cabrera-Orefice A, Guerrero-Castillo S, Diaz-Ruiz R, Uribe-Carvajal S. Oxidative phosphorylation in *Debaryomyces hansenii*: physiological uncoupling at different growth phases. Biochimie. 2014;102:124–36. 10.1016/j.biochi.2014.03.003 24657599

[66] Huh WK, Kang SO. Characterization of the gene family encoding alternative oxidase from *Candida albicans*. Biochem J. 2001;356(Pt 2):595–604. 11368790 10.1042/0264-6021:3560595 PMC1221874

[67] Rogov AG, Sukhanova EI, Uralskaya LA, Aliverdieva DA, Zvyagilskaya RA. Alternative oxidase: distribution, induction, properties, structure, regulation, and functions. Biochemistry (Mosc). 2014;79(13):1615–34. 25749168 10.1134/S0006297914130112

[68] Kern A, Hartner FS, Freigassner M, Spielhofer J, Rumpf C, Leitner L, et al *Pichia pastoris* "just in time" alternative respiration. Microbiology. 2007;153(Pt 4):1250–60. 10.1099/mic.0.2006/001404-0 17379734

[69] Georgopoulos SG, Sisler HD. Gene mutation eliminating antimycin A-tolerant electron transport in *Ustilago maydis*. J Bacteriol. 1970;103(3):745–50. 5474885 10.1128/jb.103.3.745-750.1970 PMC248153

[70] Sherald JL, Sisler HD. Antimycin A-resistant respiratory pathway in *Ustilago maydis* and *Neurospora sitophila*. Plant Physiol. 1970;46(1):180–2. 5481090 10.1104/pp.46.1.180 PMC396557

[71] Dejean L, Beauvoit B, Guerin B, Rigoulet M. Growth of the yeast *Saccharomyces cerevisiae* on a non-fermentable substrate: control of energetic yield by the amount of mitochondria. Biochim Biophys Acta. 2000;1457(1–2):45–56. 10692549 10.1016/s0005-2728(00)00053-0

[72] Spellig T, Bottin A, Kahmann R. Green fluorescent protein (GFP) as a new vital marker in the phytopathogenic fungus *Ustilago maydis*. Mol Gen Genet. 1996;252(5):503–9. 8914511 10.1007/BF02172396

[73] Xiao M, Ma J, Li H, Jin H, Feng H. Effects of hydrogen sulfide on alternative pathway respiration and induction of alternative oxidase gene expression in rice suspension cells. Z Naturforsch C. 2010;65(7–8):463–71. 20737915 10.1515/znc-2010-7-808

[74] Andronis EA, Moschou PN, Toumi I, Roubelakis-Angelakis KA. Peroxisomal polyamine oxidase and NADPH-oxidase cross-talk for ROS homeostasis which affects respiration rate in *Arabidopsis thaliana*. Front Plant Sci. 2014;5:132 10.3389/fpls.2014.00132 24765099 PMC3982065

[75] Wang J, Rajakulendran N, Amirsadeghi S, Vanlerberghe GC. Impact of mitochondrial alternative oxidase expression on the response of *Nicotiana tabacum* to cold temperature. Physiol Plant. 2011;142(4):339–51. 10.1111/j.1399-3054.2011.01471.x 21401618

[76] Vanlerberghe GC, McIntosh L. Lower growth temperature increases alternative pathway capacity and alternative oxidase protein in tobacco. Plant Physiol. 1992;100(1):115–9. 16652932 10.1104/pp.100.1.115 PMC1075525

[77] Costa JH, Jolivet Y, Hasenfratz-Sauder MP, Orellano EG, da Guia Silva Lima M, Dizengremel P, et al Alternative oxidase regulation in roots of *Vigna unguiculata* cultivars differing in drought/salt tolerance. J Plant Physiol. 2007;164(6):718–27. 10.1016/j.jplph.2006.04.001 16716451

[78] Smith CA, Melino VJ, Sweetman C, Soole KL. Manipulation of alternative oxidase can influence salt tolerance in *Arabidopsis thaliana*. Physiol Plant. 2009;137(4):459–72. 10.1111/j.1399-3054.2009.01305.x 19941623

[79] Skirycz A, De Bodt S, Obata T, De Clercq I, Claeys H, De Rycke R, et al Developmental stage specificity and the role of mitochondrial metabolism in the response of Arabidopsis leaves to prolonged mild osmotic stress. Plant Physiol. 2010;152(1):226–44. 10.1104/pp.109.148965 19906889 PMC2799359

[80] Edwards DL, Rsenberg E, Maroney PA. Induction of cyanide-insensitive respiration in *Neurospora crassa*. J Biol Chem. 1974;249(11):3551–6. 4275427

[81] Nargang FE, Adames K, Rub C, Cheung S, Easton N, Nargang CE, et al Identification of genes required for alternative oxidase production in the *Neurospora crassa* gene knockout library. G3 (Bethesda). 2012;2(11):1345–56. 23173086 10.1534/g3.112.004218 PMC3484665

[82] Dojcinovic D, Krosting J, Harris AJ, Wagner DJ, Rhoads DM. Identification of a region of the Arabidopsis AtAOX1a promoter necessary for mitochondrial retrograde regulation of expression. Plant Mol Biol. 2005;58(2):159–75. 10.1007/s11103-005-5390-1 16027972

[83] Fujita K, Kubo I. Antifungal activity of octyl gallate. Int J Food Microbiol. 2002;79(3):193–201. 12371654 10.1016/s0168-1605(02)00108-3

[84] Fujita K, Kubo I. Plasma membrane injury induced by nonyl gallate in *Saccharomyces cerevisiae*. J Appl Microbiol. 2002;92(6):1035–42. 12010543 10.1046/j.1365-2672.2002.01614.x

[85] Baumann S, Konig J, Koepke J, Feldbrugge M. Endosomal transport of septin mRNA and protein indicates local translation on endosomes and is required for correct septin filamentation. EMBO Rep. 2014;15(1):94–102. 10.1002/embr.201338037 24355572 PMC4303453

[86] Hernandez O, Garcia AM, Almeida AJ, Tamayo D, Gonzalez A, Restrepo A, et al Gene expression during activation of *Paracoccidioides brasiliensis* conidia. Yeast. 2011;28(11):771–81. 10.1002/yea.1902 21960298

[87] Walker R Jr., Saha L, Hill GC, Chaudhuri M. The effect of over-expression of the alternative oxidase in the procyclic forms of *Trypanosoma brucei*. Mol Biochem Parasitol. 2005;139(2):153–62. 10.1016/j.molbiopara.2004.11.003 15664650

[88] Johnson CH, Prigge JT, Warren AD, McEwen JE. Characterization of an alternative oxidase activity of *Histoplasma capsulatum*. Yeast. 2003;20(5):381–8. 10.1002/yea.968 12673621

[89] Xu T, Yao F, Liang WS, Li YH, Li DR, Wang H, et al Involvement of alternative oxidase in the regulation of growth, development, and resistance to oxidative stress of *Sclerotinia sclerotiorum*. J Microbiol. 2012;50(4):594–602. 10.1007/s12275-012-2015-7 22923107

[90] Zhou G, Song Z, Yin Y, Jiang W, Wang Z. Involvement of an alternative oxidase in the regulation of hyphal growth and microsclerotial formation in *Nomuraea rileyi* CQNr01. World J Microbiol Biotechnol. 2015;31(9):1343–52. 10.1007/s11274-015-1877-3 26135515

[91] Ruy F, Vercesi AE, Kowaltowski AJ. Inhibition of specific electron transport pathways leads to oxidative stress and decreased *Candida albicans* proliferation. J Bioenerg Biomembr. 2006;38(2):129–35. 10.1007/s10863-006-9012-7 17053999

[92] Avila-Adame C, Koller W. Disruption of the alternative oxidase gene in *Magnaporthe grisea* and its impact on host infection. Mol Plant Microbe Interact. 2002;15(5):493–500. 10.1094/MPMI.2002.15.5.493 12036280

[93] Avila-Adame C, Koller W. Impact of alternative respiration and target-site mutations on responses of germinating conidia of *Magnaporthe grisea* to Qo-inhibiting fungicides. Pest Manag Sci. 2003;59(3):303–9. 10.1002/ps.638 12639047

[94] Dahal K, Vanlerberghe GC. Alternative oxidase respiration maintains both mitochondrial and chloroplast function during drought. New Phytol. 2016. 10.1111/nph.14169 27579773

