# Supplementary material for: The mitochondrial alternative oxidase Aox1 is needed to cope with respiratory stress but dispensable for pathogenic development in Ustilago maydis
Source: PLoS One. 2017 Mar 8;12(3):e0173389. doi: 10.1371/journal.pone.0173389 (PMC5342259; doi:10.1371/journal.pone.0173389)
Supplement: S1 File — (PDF) [file pone.0173389.s001.pdf]

Description of *U. maydis* strains used in this study.

Strain	Locus	Progenitor strain	Short description
FB1			*Wild type strain* [1]
FB2			*Wild type strain* [1]
AB33	*b*	FB2	*Pnar:bW2bE1*, expression of active b heterodimer under control of the *nar1* promoter, strain grows filamentously upon changing the nitrogen source. [2]
FB1aox1Δ	*aox1*	FB1	Carrying a deletion of *aox1*.
FB2aox1Δ	*aox1*	FB2	Carrying a deletion of *aox1*.
AB33aox1Δ	*aox1*	AB33	Carrying a deletion of *aox1*.
FB2aox1-Gfp	*aox1*	FB2aox1Δ	Expressing Aox1 C-terminally fused to eGfp.
AB33aox1-Gfp	*aox1*	AB33aox1Δ	Expressing Aox1 C-terminally fused to eGfp.
FB2P_{otef}:aox1-Gfp	ip^S	FB2	*aox1-Gfp* is ectopically integrated in the defined ip^S locus and under control of the strong constitutive P_{otef} promoter.
FB2P_{otef}:5'UTR-aox1-Gfp	ip^S	FB2	*aox1-Gfp* is ectopically integrated in the defined ip^S locus and under control of the strong constitutive P_{otef} promoter. In this construct the native 5'UTR is retained.
AB33P_{otef}:aox1-Gfp	ip^S	AB33	*aox1-Gfp* is ectopically integrated in the defined ip^S locus and under control of the strong constitutive P_{otef} promoter.
AB33P_{otef}:5'UTR-aox1-Gfp	ip^S	AB33	*aox1-Gfp* is ectopically integrated in the defined ip^S locus and under control of the strong constitutive P_{otef} promoter. In this construct the native 5'UTR is retained.

Generation of *U. maydis* strains used in this study.

Strains	Relevant genotype	Uma	Reference	Transformed plasmid	Locus	Progenitor
AB33	*a2 P_{nar}:bW2 bE1*	133	[2]	pAB33	*b*	FB2
FB1aox1Δ	*aox1Δ*	1328	this study	pAox1Δ_HygR (pUMa2163)	*aox1*	FB1
FB2aox1Δ	*aox1Δ*	1329	this study	pAox1Δ_HygR (pUMa2163)	*aox1*	FB2
AB33aox1Δ	*aox1Δ*	1330	this study	pAox1Δ_HygR (pUMa2169)	*aox1*	AB33
FB2aox1-Gfp	*aox1-Gfp*	1333	this study	pAox1-Gfp_NatR (pUMa2169)	*aox1*	FB2aox1Δ
AB33aox1-Gfp	*aox1-Gfp*	1369	this study	pAox1-Gfp_NatR (pUMa2169)	*aox1*	AB33aox1Δ
FB2P_{otef}:aox1-Gfp	*aox1-Gfp*	1816	this study	pP_{otef}:aox1-Gfp_CbxR (p2768)	*ip^S*	FB2
FB2P_{otef}:5'UTR-aox1-Gfp	*aox1-Gfp*	1814	this study	pP_{otef}:5UTR-aox1-Gfp (p2767)	*ip^S*	FB2
AB33P_{otef}:aox1-Gfp	*aox1-Gfp*	1817	this study	pP_{otef}:aox1-Gfo_CbxR (p2768)	*ip^S*	AB33
AB33P_{otef}:5'UTR-aox1-Gfp	*aox1-Gfp*	1815	this study	pP_{otef}:5UTR-aox1-Gfp (p2767)	*ip^S*	AB33

Description of plasmids used for *U. maydis* strain generation.

Plasmid	pUMa	Resistance cassette	Short description
pAox1Δ_HygR	2163	SfiI-insert of MF1hs	Plasmid for generating deletion mutants of *aox1*. Resistance cassette is flanked by 1 kb upstream and 1 kb downstream region of *aox1*. Flanking regions were amplified by PCR using oRL1400/oRL1401 and oRL1402/oRL1403 and UM521 wild-type DNA as template. Plasmid was generated by Golden Gate cloning [3]
pAox1-Gfp_NatR	2169	SfiI-insert of pMF5-1n	Plasmid for generating eGfp fusions of *aox1*. A cassette containing Gfp, the T_{nos} terminator and a nourseothricin resistance cassette is flanked by 2.4 kb upstream region including 1.3 kb of the *aox1* ORF and 1 kb downstream region of *aox11*. Flanking regions were amplified by PCR using oRL1400/oRL1425 and oRL1402/oRL1403 and UM521 wild-type DNA as template.
pP_{otef}:5UTR-aox1-Gfp	2767	CbxR for integration at *ip*^{S} locus	Plasmid for ectopical integration and expression of *aox1-Gfo*. Contains 1.3 kb open reading frame (ORF) of *aox1* N-terminally fused with eGfp flanked by strong constitutively active promoter P_{otef} and transcriptional terminator T_{nos} upstream and downstream, respectively. This construct retains the 65 bp 5‘ UTR of *aox1*. The ORF and 5' UTR were amplified by PCR using oMF894/oDD808 and pAox1-Gfp_NatR as template.
pP_{otef}:aox1-Gfp_CbxR	2768	CbxR for integration at *ip*^{S} locus	Plasmid for ectopical integration and expression of *aox1-Gfp*. Contains 1.3 kb ORF of *aox1* N-terminally fused with eGfp flanked by strong constitutively active promoter P_{otef} and transcriptional terminator T_{nos} upstream and downstream, respectively. The ORF were amplified by PCR using oMF894/oDD809 and pAox1-Gfp_NatR as template.

DNA oligonucleotides used in this study.

Designation	Nucleotide sequence (5' --> 3')	Remarks
oRL1400	GGTCTCGCCTGCAATATTCCACTGAGATAGTCGTTGAGG	*aox1* u2
oRL1401	GGTCTCCAGGCCGGTTACTGGCTTGGGCTG	*aox1* u3
oRL1402	GGTCTCCGGCCCTGCTTTCCAACTGGATTCG	*aox1* d1
oRL1403	GGTCTCGCTGCAATATTTTTCCCATGAGATGCTGC	*aox1* d2
oRL1425	AATGGCCGCGTTGGCCGCAGCGGTCTTTTCGGCCGC	*aox1* u3-fus
oMF894	TCGCAAGACCGGCAACAG	
oDD808	GGCGAATTCCAGACTTTTAGCAACCATACCAAAGC	Ectopic with 5'UTR
oDD809	CGGGAATTCATGTACGTTAGTACGCCCATC	Ectopic without 5'UTR
oRL1399	GTTCAACACGTCCGGAGG	*aox1* u1
oRL1404	CGCTGTTGCTCCATTCGG	*aox1* d3
oRL1405	AGGTACCCGGACCACAAC	*aox1* p1
oRL1406	GATTGAGCCAACCGTCGG	*aox1* p2

Supplementary references

1. Banuett F, Herskowitz I. Different a alleles of *Ustilago maydis* are necessary for maintenance of filamentous growth but not for meiosis. Proceedings of the National Academy of Sciences of the United States of America. 1989;86(15):5878-82.

2. Brachmann A, Weinzierl G, Kamper J, Kahmann R. Identification of genes in the bW/bE regulatory cascade in *Ustilago maydis*. Molecular microbiology. 2001;42(4):1047-63.

3. Terfruchte M, Joehnk B, Fajardo-Somera R, Braus GH, Riquelme M, Schipper K, et al. Establishing a versatile Golden Gate cloning system for genetic engineering in fungi. Fungal genetics and biology : FG & B. 2014;62:1-10.
