# Supplementary figures and images for: The mitochondrial alternative oxidase Aox1 is needed to cope with respiratory stress but dispensable for pathogenic development in Ustilago maydis
Source: PLoS One. 2017 Mar 8;12(3):e0173389. doi: 10.1371/journal.pone.0173389 (PMC5342259; doi:10.1371/journal.pone.0173389)

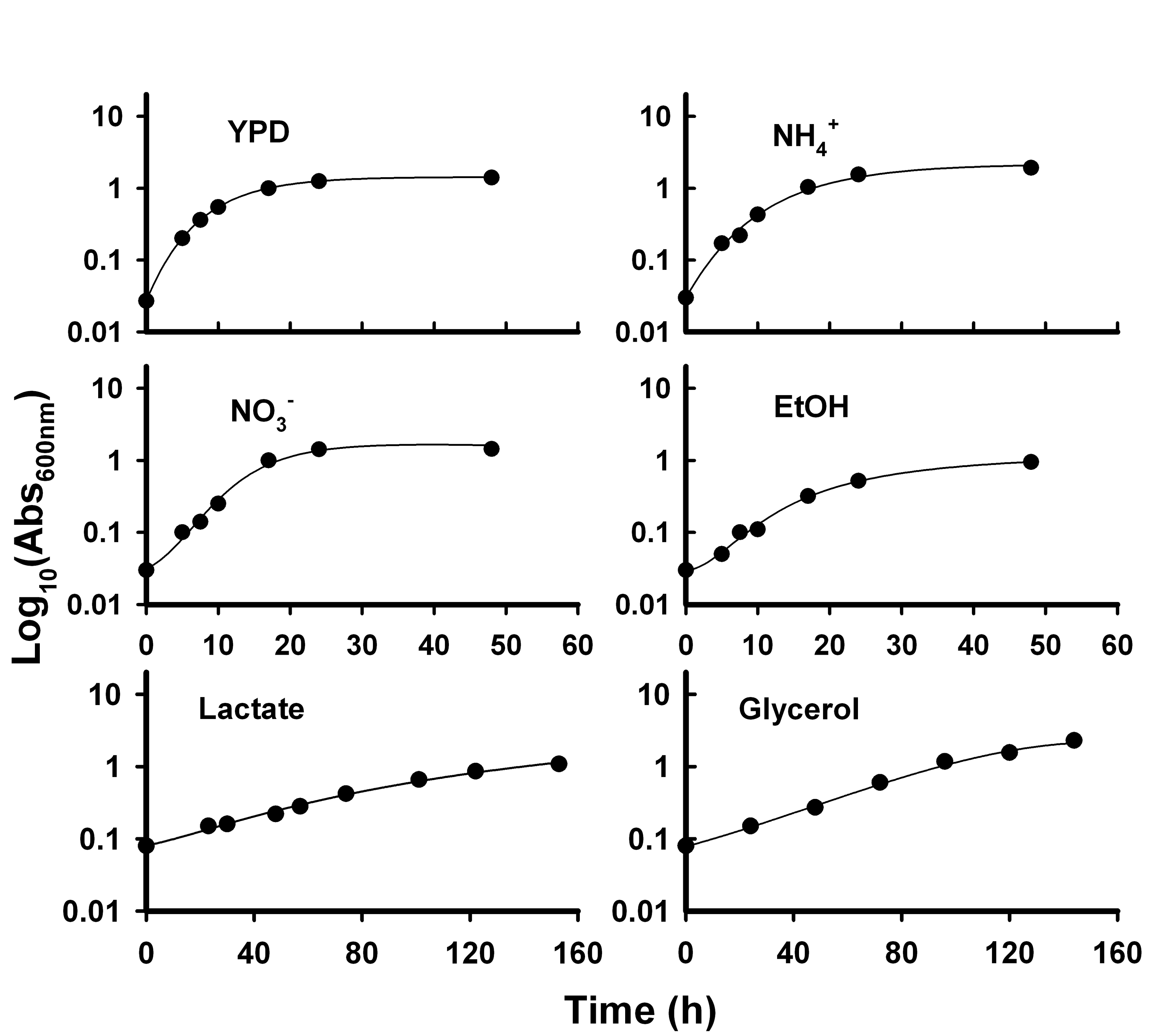

Supplement: S1 Fig — Ustilago maydis was grown in different carbon and nitrogen sources and the absorbance at 600nm measured at the indicated time. YPD, rich medium (1.0% glucose, 0.25% peptone, and 0.5% yeast extract); NH4+, minimal medium with glucose (1.0%) and ammonium sulfate (0.3%); NO3-, minimal medium with glucose (1.0%) and potassium nitrate (0.3%); EtOH, minimal medium with ethanol (0.4%) and ammonium sulfate (0.3%); Lactate, minimal medium with lactate (1.0%) and ammonium sulfate (0.3%); Glycerol, minimal medium with glycerol (1.0%) and ammonium sulfate (0.3%). (TIF) [file pone.0173389.s002.tif]

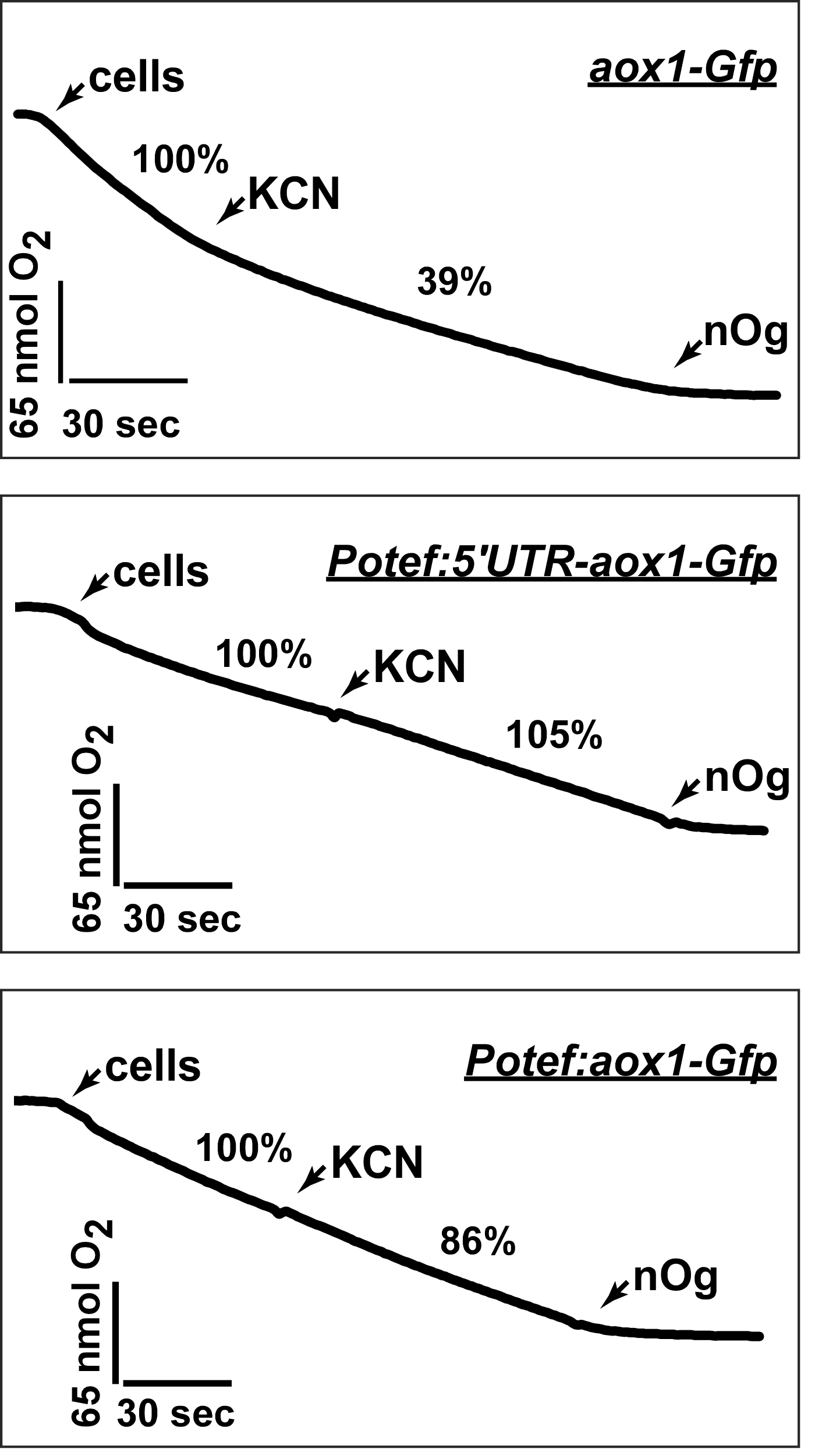

Supplement: S2 Fig — Oxygen consumption was measured as indicated under material and methods. Respiratory traces of aox1-Gfp, Potef:5’UTR-aox1-Gfp, and Potef:aox1-Gfp sporidia in the stationary phase. Arrows show the addition of KCN and n-octylgallate (nOG). (TIF) [file pone.0173389.s003.tif]

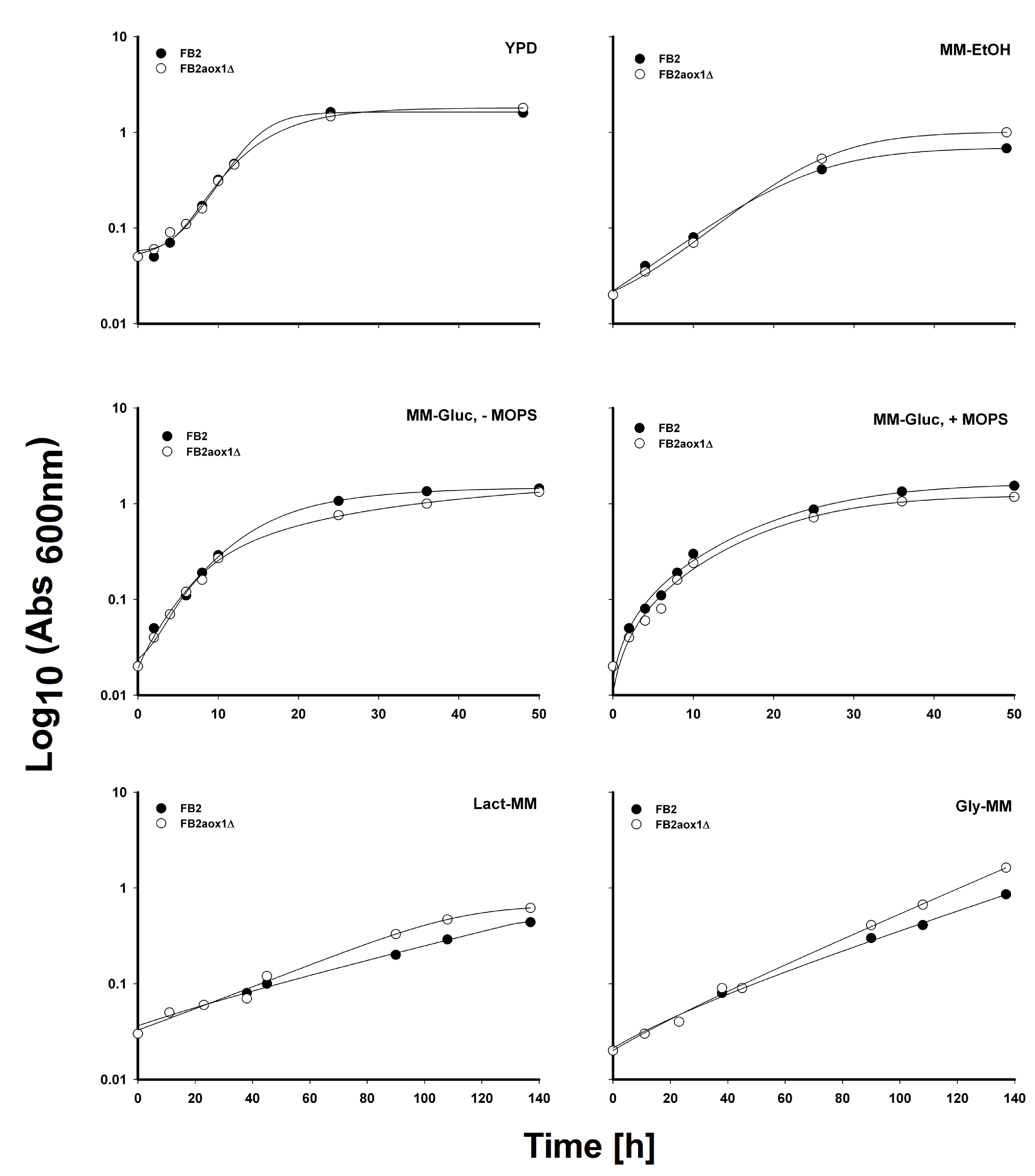

Supplement: S3 Fig — Ustilago maydis wild type and the strain lacking aox1 were grown in different carbon sources as in S1 Fig, using ammonium sulfate (0.3%) as nitrogen source. Minimal medium with glucose was prepared with or without 50 mM MOPS. (TIF) [file pone.0173389.s004.tif]
